# Efficacité et tolérance de l'azathioprine lors du traitement de base de la sclérose en plaques: cas du Maroc

**DOI:** 10.11604/pamj.2024.49.16.38051

**Published:** 2024-09-18

**Authors:** Ali Mirhani, Nabila Auajjar, Chouki Slimani, Benaissa Attarassi

**Affiliations:** 1 Faculté des Sciences, Université Ibn-Tofail, Kenitra, Maroc,; 2 Centre Hospitalier Universitaire, Service de Neurologie, Hôpital El Idrissi, Kenitra, Maroc

**Keywords:** Azathioprine, enquête-rétrospective, sclérose en plaques, score EDSS, Maroc, Azathioprine, retrospective survey, multiple sclerosis, expanded disability status scale score, Morocco

## Abstract

**Introduction:**

notre étude vise à évaluer l'efficacité de l'azathioprine comme traitement de base chez les patients atteints de la sclérose en plaques (SEP) rémittente récurrente ou de la SEP progressive, qui auraient dû prendre les interférons bêta, mais par manque de moyen ont pris l´azathioprine.

**Méthodes:**

parmi 31 patients, 17 étaient atteints d´une SEP récurrente-rémittente (SEP-RR), 11 atteints d´une SEP progressive primaire (SEP-PP) et 3 atteints d´une SEP secondairement progressive (SEP-SP). Les patients ont reçu l´azathioprine, à raison de 3 mg/kg/j par voie orale sur une période de 2 ans. L´effet d´azathioprine a été évalué par le score d´Expanded Disability Status Scale (EDSS). Le test de comparaison des moyennes nous a permis de déterminer la performance du traitement.

**Résultats:**

dans le cas du SEP-RR, la note moyenne de l'EDSS se situe entre 4,2 et 3,6 ± 1,4. Quatre-vingt-deux virgule quatre pour cent des patients étaient stables ou en amélioration avec une différence très hautement significative (P=0,000). Pour la SEP-PP, le résultat moyen de l'EDSS reste constant et correspond à 5,7 ± 0,4. Cinquante-quatre virgule cinq pour cent des patients ont affiché une stabilité ou une amélioration, avec une différence hautement significative (P=0,005). Dans le cas de SEP-SP, la note moyenne de l'EDSS se situe entre 4,5 et 4,1 ± 1,3. Soixante-six virgule sept pour cent des patients étaient stables ou améliorés par une différence non significative (P= 0,1).

**Conclusion:**

les résultats de cette étude confirment que l'azathioprine est bénéfique dans le traitement des différents types de la sclérose en plaques au Maroc.

## Abstract (en)

**Introduction:**

the purpose of our study is to evaluate the efficacy of azathioprine as first-line treatment in patients with relapsing-remitting multiple sclerosis (RRMS) or progressive multiple sclerosis (PMS), who were supposed to be treated with beta-interferons but, due to limited resources, received azathioprine instead.

**Method:**

among the 31 patients, 17 had relapsing-remitting MS (RRMS), 11 had primary progressive MS (PPMS), and 3 had secondary progressive MS (SPMS). Patients received azathioprine orally at a dose of 3 mg/kg/day over 2 years. The effect of azathioprine was assessed using the Expanded Disability Status Scale (EDSS) score. Compare means t-test was used to determine the treatment performance.

**Results:**

for RRMS, the mean EDSS score ranged between 4.2 and 3.6 ± 1.4. A total of 82.4% of patients were stable or improved, with a very highly significant difference (P= 0.000). For PPMS, the mean EDSS score remained constant at 5.7 ± 0.4. A total of 54.5% of patients showed stability or improvement, with a highly significant difference (P= 0.005). In the case of SPMS, the mean EDSS score ranged between 4.5 and 4.1 ± 1.3. A total of 66.7% of patients were stable or improved but with a non-significant difference (P= 0.1).

**Conclusion:**

the results of this study confirm that azathioprine is beneficial in the treatment of different types of multiple sclerosis in Morocco.

## Introduction

On entend par sclérose en plaques (SEP), une maladie inflammatoire chronique du système nerveux central qui provoque des lésions axonales précoces et entraîne un handicap irréversible chez de nombreux patients [1]. Il est maintenant bien admis que cette atteinte neurologique irréversible se manifeste lorsqu'un seuil de perte axonale chronique est atteint et que les ressources compensatoires du SNC sont épuisées [1]. Malgré les modèles cliniques existants, le développement du SEP et ses caractéristiques cliniques diffèrent significativement d'une personne à l'autre [2]. La prévalence du SEP a été estimée à 30 pour 100 000 personnes dans le monde en 2008 [3], ce qui en fait l'une des affections neurologiques du système nerveux central les plus courantes. Cette prévalence mondiale a augmenté d'environ 33 cas pour 100 000 personnes en 2013 [3]. Quatre sous-types principaux de la SEP sont révélés par les descriptions phénotypiques de la maladie à partir de 1996: la forme récurrente-rémittente (RR), la forme secondaire progressive (SP), la forme primaire progressive (PP) et la forme récurrente progressive (RP) [2].

La forme RP a toutefois été supprimée à la suite d'une révision en 2013 [4]. La SEP-RR est la plus répandue de ces sous-types et représente 85% à 90% des cas au début de la maladie [5]. Les neurologues considèrent le traitement par l'azathioprine comme une nouvelle approche thérapeutique parmi les thérapies. Depuis plus de 30 ans, l'azathioprine (AZA), qui est un analogue de la purine, est utilisée pour traiter la sclérose en plaques [6]. En effet, dans de nombreux pays, on utilise l'azathioprine comme traitement du SEP sur la base d'essais contrôlés randomisés (ECR) par rapport au placebo [7]. Cette azathioprine est un médicament immunosuppresseur et cytotoxique qui fonctionne comme un précurseur de la mercaptopurine; un inhibiteur de la synthèse de l'ADN [8]. Par conséquent, il a un effet plus fort sur les cellules en prolifération comme les lymphocytes T et B du système immunitaire [8]. Ceci s´explique par l'antimétabolite AZA, qui est le promédicament de la 6-mercaptopurine (6-MP), qui bloque la synthèse des nucléotides puriques [9].

Le thioguanosine triphosphate et l'acide thioinosinique, entre autres produits métaboliques de l'AZA, fonctionnent comme de “faux” substrats et entravent la réplication de l'ADN [9]. Malgré le fait que l'utilisation de l'azathioprine dans la SEP soit fortement limitée en raison du manque de données provenant d'essais cliniques répondant aux normes de qualité actuelles [10,11], l'amélioration de l'état de certains patients suite à un traitement avec ce médicament a conduit à son utilisation systématique. Malgré l´accord unanime en Europe, l'AZA est approuvée comme traitement de seconde ligne de la SEP-RR après l'échec des interférons bêta (IFN) ou en cas de stabilité clinique lors d'un précédent traitement à l'AZA; mais il n'est pas approuvé pour le traitement de la SEP aux États-Unis [9].

De nombreuses études ont été réalisées sur l´efficacité et la tolérance d´azathioprine. Dans les études portant sur l'AZA, les différentes doses d'AZA utilisées, étaient comprises entre 2 et 4,4 mg/kg de poids corporel, avec une durée qui allait de 18 mois à 3 ans [6]. Les résultats du traitement à l'azathioprine chez des patients marocains atteints de la SEP-RR, SEP-PP et SEP-SP qui n'avaient jamais eu de traitement de fond auparavant sont rapportés dans cette étude. L'objectif est de comparer les différentes réactions de chacune des trois formes de la SEP afin d'évaluer l'efficacité et la tolérance de l'azathioprine sur une période de deux ans.

## Méthodes

**Conception de l´étude:** une analyse rétrospective de cohorte a été réalisée pour étudier l'efficacité de l'azathioprine en tant que traitement de fond pour les patients atteints de la sclérose en plaques (SEP) récurrente-rémittente ou progressive qui auraient autrement dû prendre des interférons bêta mais par manque de moyen ont pris l'azathioprine.

**Population étudiée:** trente-et-un (31) patients atteints de SEP-RR, SEP-PP et SEP-SP ont été inclus dans l'étude. Ils ont été admis à l'hôpital El Idrissi de Kenitra Maroc, et au Centre Hospitalier Universitaire (CHU) entre juillet 2009 et juillet 2019.

**Critères d´inclusion et exclusion de l´étude:** l'IRM du patient doit établir un diagnostic de la sclérose en plaques conformément aux critères de McDonald *et al*. 2010 [12] et de Poser *et al*. [13] pour que le patient puisse être admis à l'étude. L'essai a exclu les patients qui avaient reçu un autre traitement de fond ou qui présentaient une contre-indication à l'azathioprine. Chaque femme a subi un examen gynécologique complet, et celles qui étaient fertiles ont eu une contraception fiable.

**L´évaluation par le score *Expanded Disability Status Scale* (EDSS):** les patients ont été suivis et évalués durant toute la période du traitement de l'étude (2 ans). L´effet d´azathioprine a été évalué par le score moyen de l'EDSS [14]. L´EDSS initial est déterminé avant le traitement tandis que l´EDSS final est déterminé après 24 mois du traitement. Le test de comparaison des moyennes nous a permis de déterminer la performance du traitement, et les patients aggravés auront une augmentation de 0,5 point au-dessus de 5,5 du score EDSS moyen initial et les patients stables ou améliorés auront une diminution d´au moins 1 point au-dessous de 5,5 du score EDSS moyen initial. A la fin du traitement, le neurologue a évalué le score EDSS moyen (EDSS moyen final) et l'a comparé avec le score EDSS moyen initial. Le suivi pendant la visite dépendait de l'évaluation clinique, de l'EDSS et les analyses de sang (hématologie et biochimie).

**Variables étudiées**: les principales variables étudiées étaient: le nombre de patients, le sexe, l´âge au début de la maladie, l´âge au début du traitement, la durée de l´évolution de la maladie avant l´utilisation du traitement par l´azathioprine, la durée du traitement, l’EDSS moyen initial, l’EDSS moyen final et les effets indésirables du traitement.

### Ressource de données et mesure

**Outil de collecte de données:** comme méthode de collecte de données, un questionnaire standardisé étayant les détails démographiques et cliniques des patients SEP admis au CHU de Kenitra entre juillet 2009 et juillet 2019 a été adopté.

**Collecte de données:** les caractéristiques démographiques révèlent le sexe du patient, l´âge au début de la maladie, l´âge au début du traitement, la durée de la progression de la maladie avant l'utilisation de l'azathioprine comme traitement, la durée du traitement et le nombre de patients. Quant aux caractéristiques cliniques, ils comportent l´EDSS moyen initial et l´EDSS moyen final. Un questionnaire a été utilisé pour recueillir toutes les données démographiques et cliniques. Ce questionnaire a été pré-testé à l´aide d´un échantillon de 23% de la population de la région de Kenitra. Un questionnaire structuré a été utilisé pour la collecte des données auprès du neurologue par le biais d´un entretien. Les données démographiques et cliniques ont été téléchargées et intégrées dans une base de données. Cette dernière était conservée sur un ordinateur protégé par un mot de passe.

**L´analyse des données:** un logiciel de statistique des sciences sociales a été utilisé pour recueillir et évaluer les données (SPSS). La médiane ou la moyenne (écart type) des variables quantitatives ont été utilisées pour les exprimer, selon la dispersion des résultats. Les variables qualitatives ont été exprimées en termes de fréquences et de pourcentages. La matrice Anova et le test T de Student ont été utilisés pour comparer les scores moyens EDSS initiaux et finaux pour les types progressif primaire, progressif secondaire et récurrent-rémittent, tandis que le Khi2 et le R de Pearson ont été utilisés pour évaluer l'évolution du handicap. Pour P< 0,05, la différence est considérée comme significative.

**Considération éthique:** le comité d'éthique du service provincial a approuvé cette étude (N° 4361 du 21 octobre 2019). Afin d'offrir aux patients l'autonomie et la capacité de prendre leurs propres décisions, l'étude établit les conditions essentielles à la pratique du consentement libre et éclairé. Tous les règlements essentiels au respect des patients représentants des difficultés de lecture sont assurés également pour la protection de leurs intérêts. Afin de protéger la confidentialité des données personnelles, tous les outils nécessaires à la préservation de la vie privée et de renseignement personnel sont également réunis.

## Résultats

Les caractéristiques examinées pour la cohorte de patients marocains atteints de la SEP étaient le nombre de patients, le sexe, l'âge au début de la maladie, l'âge au début du traitement, la durée de progression de la maladie avant le début du traitement à l'azathioprine et la durée du traitement. En outre, nous avons aussi déterminé le score EDSS moyen final, par rapport au score EDSS moyen initial.

**Analyse démographiques et cliniques des patients atteints de la sclérose en plaques:** dans cette analyse rétrospective de cohorte, l'azathioprine a été administrée à un total de 31 patients, dont 55% avaient une SEP-RR, 35% une SEP-PP et 10% une SEP-SP. La SEP-RR a été présenté par 17 patients, dont 11 femmes et 6 hommes. Au début de la maladie, l´âge moyen était de 30 ± 8ans (Tableau 1). De même, au début du traitement l´âge moyen était de 35 ± 7 ans. Nous précisons que la maladie a duré en moyenne 4 ans avant l´utilisation du traitement (fourchette: 1;10), et le traitement par l´azathioprine a duré en moyenne 2 ans (fourchette : 1;2). Le score EDSS initial était en moyenne de 4,2 ± 1,4. La SEP-PP a été présenté par 11 patients dont 10 femmes et 1 homme. Au début de la maladie, l´âge moyen était de 39 ± 3 ans (Tableau 1). De même, au début du traitement l´âge moyen était de 43 ± 3 ans. La maladie a duré en moyenne 3 ans avant le début du traitement (fourchette: 2;8), et le traitement à son tour a duré en moyenne 2 ans (fourchette: 1;2). Le score EDSS initial moyen était de 5,7 ± 0,4. Enfin, la SEP-SP a été présentée par 3 patients dont 2 femmes et 1 homme. Au début de la maladie, l´âge moyen était de 46 ± 6 ans (Tableau 1). Au début du traitement, l´âge en moyenne était de 48 ± 5,2 ans. La maladie a duré en moyenne 2 ans avant le début du traitement (fourchette: 1;3), et le traitement a duré en moyenne 2 ans (fourchette: 1;2). Le score EDSS initial moyen était de 4,5 ± 1,3.

**Tableau 1:** caractéristiques démographiques et médicales des patients atteints de la sclérose en plaques-récurrente-rémittente

Caractéristiques	SEP-RR	SEP-PP	SEP-SP
Nombre de patients	17 (55%)	11 (35%)	3 (10%)
Sex-ratio (F/H)	1,8 (11/6)	10 (10/1)	2 (2/1)
Age moyen au début de la maladie (année) ±6	30 ±8	39 ±3	46
Age moyen au début du traitement (année) ±5,2	35 ±7	43 ±3	48
Durée de la maladie: médiane (année)	4 (1; 10)	3 (2; 8); 2 (1; 3)	
Durée du traitement: médiane (année)	2 (1; 2)	2 (1; 2); 2 (1; 2)	
EDSS moyen initial	4,2 ±1,4	5.7 ±0,4; 4.5 ±1.3	
EDSS moyen final	3,6 ±1,8	5.7 ±0.9; 4.1 ±1	

SEP-RR: sclérose en plaques récurrente-rémittente; SEP-PP: sclérose en plaques primairement progressive et SEP-SP: sclérose en plaques secondairement progressive

**Figure 1:**
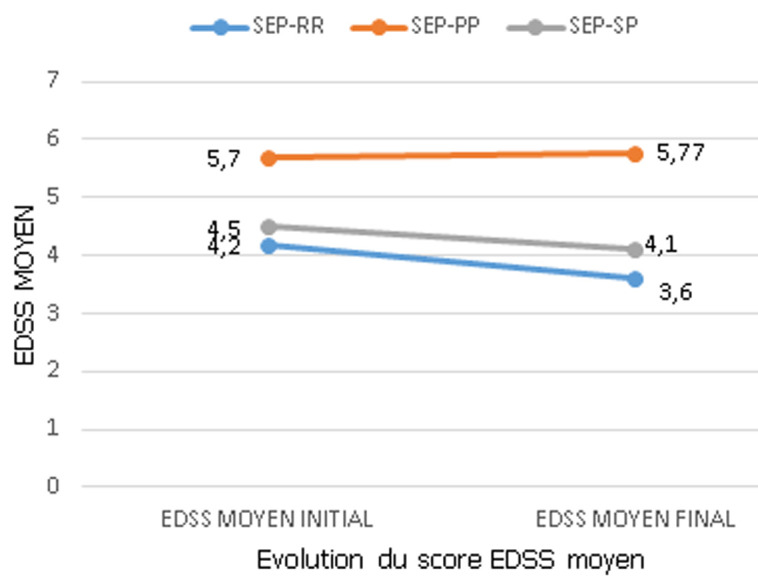
évolution du Score Expanded Disability Status Scale Score moyen pendant la surveillance des patients atteints de la sclérose en plaques (SEP)

**Analyse du score EDSS moyen au cours du suivi des patients atteints de la sclérose en plaques:** nous avons observé chez les patients atteints d´une SEP-RR et SEP-SP une très bonne amélioration du score EDSS final moyen. Nous avons également noté chez les patients atteints d´une SEP-PP, une stabilisation du score EDSS final (Figure 1). Le score EDSS moyen initial de 4,2 pour les personnes atteintes de la SEP-RR a été diminué de 0,6 pour atteindre un score EDSS moyen final de 3,6. En revanche, chez les patients atteints de la SEP-PP, le score EDSS moyen initial de 5,7 est resté le même lors du score EDSS moyen final. Enfin, chez les malades atteints de la SEP-SP, le score EDSS moyen initial de 4,5 a baissé de 0,4 à 4,1 au score EDSS moyen final (Figure 1). Les moyennes du score EDSS initial et final du forme SEP-RR ont montré une différence très hautement significative (P= 0,000) lors de l'analyse statistique. Chez la forme SEP-PP, la différence était hautement significative (P= 0,005) entre les moyennes du score EDSS initial et final lors de l'analyse statistique. Néanmoins, lors de l'analyse statistique chez la forme SEP-SP, une différence non significative (P= 0,1) entre les moyennes du score EDSS initial et final a été montré (Figure 1).

**Figure 2:**
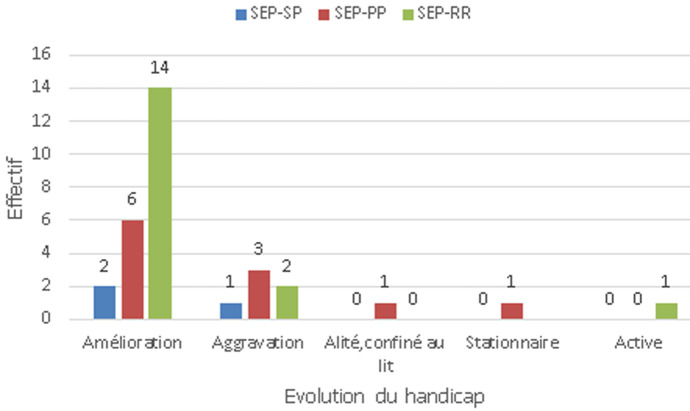
évolution du handicap des patients avec sclérose en plaques-récurrente-rémittente (SEP-RR) et sclérose en plaques progressive

**Figure 3:**
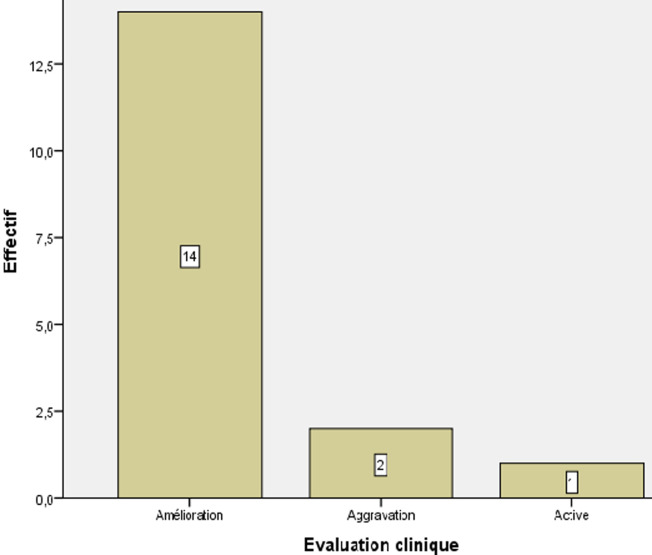
évolution du handicap chez les patients souffrant de la sclérose en plaques-récurrente-rémittente (SEP-RR)

**Figure 4:**
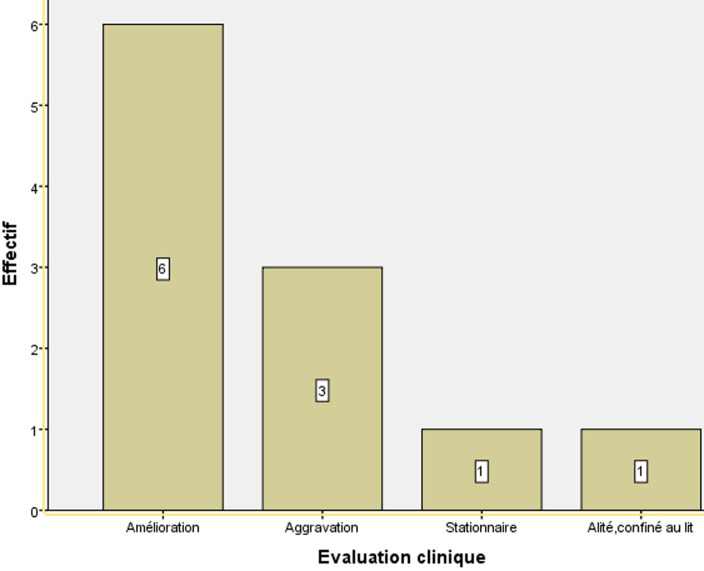
évolution du handicap chez les patients atteints de la sclérose en plaques-primaire progressive (SEP-PP)

**Analyse du handicap des patients atteints d´une sclérose en plaques:** chez les patients atteints par une SEP-RR, l´aggravation était notée chez 11,8% alors que les autres patients étaient stables, améliorés ou actifs. Par contre, chez les patients atteints par une SEP-PP, l´aggravation était notée chez 27,3% alors que les autres patients étaient stables, améliorés, stationnaires ou alités, confinés au lit. Enfin les patients atteints par une SEP-SP, l´aggravation était notée chez 33,3% tandis que les autres patients ont connu une stabilité ou une amélioration (Figure 2). Ainsi, au stade final de l´étude, 82,4% (14/17) des patients de la SEP-RR étaient stables ou améliorés. 54,5% (6/11) des patients de la SEP-PP étaient stables ou améliorés à la fin de l´étude. Et 66,7% (2/3) des patients de la SEP-SP étaient stables ou améliorés aussi à la fin de l´étude (Figure 2, Figure 3, Figure 4). Il est à noter que la comparaison de la réponse thérapeutique au stade final de l´étude, a révélé que la réponse était très hautement significative chez la forme SEP-RR. Elle est hautement significative chez la forme SEP-PP. Par contre, chez la forme SEP-SP, elle est non significative (Figure 1). Nous constatons que le traitement est bien toléré chez tous les patients. Aucun patient ne souffrait de contre-indications.

## Discussion

Les résultats de cet essai de cohorte qui incluait des patients présentant une SEP-RR, une SEP-PP et une SEP-SP, et qui n'avaient pas pris d'immunomodulateurs ou d'immunosuppresseurs auparavant, démontrent que l'azathioprine a un effet bénéfique sur l'évolution du handicap qui se trouve stabilisé ou amélioré chez plus de 50% des patients. Les patients avec SEP-RR semblaient mieux répondre que ceux avec SEP-SP et SEP-PP avec une fréquence estimée d'amélioration ou de stabilisation de 82,4% contre 66,7% et 54,5%, respectivement. En outre, le score EDSS initial moyen des patients SEP-RR a également diminué, passant de 4,2 à 3,6 au score EDSS final moyen. Par contre, chez les patients du SEP-PP, le score EDSS moyen de départ de 5,7 est demeuré le même que le score EDSS moyen final. Enfin, chez les patients atteints de la sclérose en plaques-SP, le score moyen initial de l'EDSS a diminué de 4,5 à 4,1 du score moyen final de l'EDSS. La variation entre les moyennes du score EDSS initial et final à son tour, était très hautement significative chez la forme SEP-RR. Elle est hautement significative chez la forme SEP-PP. Par contre, elle a été non hautement significative chez la forme SEP-SP.

Comme il n'existe aucune thérapie curative de la SEP, le traitement de la SEP vise à réduire le risque d'évolution de la maladie, le risque de rechutes et de progression du handicap [15]. L'AZA est un immunosuppresseur non spécifique censé inhiber principalement les immunocytes immatures [16]. Cet immunosuppresseur est connu pour son antagonisme sur le métabolisme des purines [17]. L'antimétabolite AZA est le promédicament de la 6-mercaptopurine (6-MP), qui agit par blocage de la synthèse des nucléotides puriques [9]. D'autres métabolites de l´AZA, tels que le thioguanosine triphosphate et l'acide thioinosinique, interfèrent avec la réplication de l'ADN en tant que “faux” substrats [9]. L'azathioprine est utilisée comme traitement de la SEP dans de nombreux pays à partir d'essais contrôlés randomisés (ECR) contre le placebo, il y a plus de deux décennies [7]. Il est approuvé en Europe comme traitement de deuxième intention du SEP-RR suite à une défaillance de l'interféron (IFN) bêta. Toutefois, ce n'est pas pareil aux États-Unis; l'AZA n'a pas trouvé son authenticité dans le traitement de la SEP [9].

Pour une raison d´insuffisance de données résultant aux normes de qualité actuelle, l'utilisation de l'AZA dans la SEP est fortement limitée [10,11]. Dans de nombreux cas, la réponse aux traitements à base d´immunomodulateurs ou d´immunosuppresseurs utilisée dans la SEP n'était pas satisfaisante et récemment, on fait recours à de nouvelles approches thérapeutiques. Lus et al. [18], avaient étudié l'efficacité clinique, la sécurité et la tolérance du traitement combiné entre AZA et IFN-beta (1a) chez 23 patients atteints de la SEP-RR cliniquement définie et qui n'avaient pas répondu auparavant à l'une ou l'autre des monothérapies. Après 2 ans de traitement, cette thérapie combinée semble être sûre et bien tolérée et aucun effet secondaire grave n'a été signalé [18]. En outre, l´association de l'AZA avec un traitement IFN a été aussi évaluée sur 15 patients, les données suggèrent des effets d'imagerie positifs et Il n'y a eu aucune réaction indésirable grave. Néanmoins 20% des patients ont mis fin au traitement en raison d'effets secondaires [19].

En cas de comparaison entre l'IFN-bêta et l'AZA, l'AZA a montré une non-infériorité quant au taux de rechute annualisé (ARR) chez 150 patients atteints de la SEP qui ont été randomisés pour recevoir soit l'IFN-bêta, soit l'AZA [20]. Les différents résultats d'imagerie sont restés en dessous de la marge de non-infériorité, ce qui indique une non-infériorité par rapport à l'IFN-bêta par une efficacité d'au moins 73% [20]. Egalement une comparaison de ces deux médicaments a été effectuée dans une méta-analyse indirecte en ce qui concerne le taux de rechute à 24 mois, sur les patients présentant la SEP sous toutes ses formes, pour lesquelles aucune infériorité n'a encore été constatée. Au contraire, l'AZA est apparue être plus efficace que l'IFN-bêta avec un Risque Relatif (RR) de 0,88 (IC 95%: 0,78 à 1,08) [21]. Toutefois, les patients de notre étude étaient tous sans traitement de base, recevant l'azathioprine en tant que premier traitement, d'où l´importance de notre étude. En effet, l'azathioprine est à utiliser dans la SEP car elle a une certaine efficacité [22].

Selon une étude réalisée par Casetta *et al*. en 2007, en cinq essais contenant des données provenant de plus de 400 patients traités par l´azathioprine sur une période de 3 ans, les résultats décrivent des réductions de l'ARR de 20%, 23% et 18% dans la première, la deuxième et la troisième année respectivement [22]. Dans une méta-analyse chez des patients, des essais utilisant l'AZA par voie orale (2-3 mg/kg/jour), la probabilité de ne pas subir de rechute a été évaluée: les rapports de cohortes attribuables à l'AZA à 1, 2 et 3 ans étaient de 1,5, 2,0 et 2,0, respectivement [23]; Palace *et al*. [24] ont par ailleurs confirmé ces données, et ont montré une proportion croissante de patients qui ne présentaient pas de rechute au bout de 2 ans. Malgré le succès de l´azathioprine dans le traitement de la SEP sur toutes les formes détaillées ci-dessus, on peut observer une leucopénie, une anémie macrocytaire et des anomalies dans la fonction hépatique durant le traitement à l'azathioprine [11].

L'essai britannique et hollandais montre que la leucopénie devient généralement moins fréquente avec le temps, 26% des patients étaient leucopéniques à la fin de la première année, tandis que seulement 8% étaient leucopéniques à la fin de la troisième année [25]. Néanmoins, des mesures contraceptives sont régulièrement recommandées pour les femmes et les hommes recevant un traitement à l'AZA, ce dernier peut toutefois continuer à être utilisé pendant la grossesse (après une évaluation minutieuse des risques et des avantages) et qu'il n'y a aucune contre-indication absolue à son usage [6]. Une autre étude montre une vaste expérience sur la combinaison de l'AZA et la contraception chez les hommes et les femmes même en gestation, notamment en gastro-entérologie [26]. L´étude est limitée à la province de Kenitra (23% de la population régionale), avec une collecte de données limitée au CHU. Tous les patients n´ont été traités que par l´AZA sans recours aux autres traitements types interférons ou Acétate de Glatiramère (AG) ou cyclophosphamide; on ne peut alors se prononcer définitivement sur l´efficacité de l´AZA considérée seule.

## Conclusion

Les résultats obtenus montrent l'effet bénéfique de l'azathioprine dans le traitement de base des différentes formes de la SEP au Maroc. Une très bonne amélioration du score EDSS final moyen est observée chez les patients ayant une SEP-RR et SEP-SP. Par contre, chez les patients atteints de la sclérose en plaques-PP, on observe une stabilisation du résultat final moyen de l'EDSS. En outre, les patients avec la SEP-RR répondent au traitement de manière plus favorable que ceux avec la SEP-PP et la SEP-SP. Finalement, grâce à notre étude, nous pouvons affirmer que l'azathioprine pourrait être un traitement de premier recours sur les différentes formes de la SEP. Cependant, des essais cliniques supplémentaires doivent être réalisés pour démontrer l'importance de cet immunosuppresseur dans le traitement des patients de la SEP, afin de mieux déterminer le profil des répondants et améliorer la prise en charge thérapeutique des patients atteints de la SEP.

### Etat des connaissances sur le sujet

- Il n´existe aucune thérapie curative de la SEP, le traitement de la SEP est conçu pour réduire le risque que la maladie évolue, le risque de la rechute et de progression du handicap;
- L´AZA est utilisé dans le traitement de la SEP depuis plus de 30 ans;
- Il est approuvé qu´en Europe l´AZA est considérée comme traitement de seconde ligne de la SEP-RR après l´échec des interférons beta (IFN) ou en cas de stabilité clinique lors d´un précédent traitement à l´AZA; néanmoins, elle n´est pas approuvé pour le traitement de la SEP aux États-Unis.

### Contribution de notre étude à la connaissance

- L´AZA permet une bonne amélioration et une stabilité du score EDSS moyen final chez les patients de la SEP, malgré le non recours aux interférons; cela est en faveur de la diminution du coût des soins;
- Les patients souffrant de la SEP, étaient traités par l´AZA comme traitement de fond sans aucun autre traitement au préalables;
- Les résultats obtenus dans cette étude, mettent en évidence l'effet bénéfique de l'AZA sur l´amélioration du handicap chez des patients atteints par les différentes formes de la SEP.

## Remerciements

Nous remercions tous ceux qui ont fourni de l'aide dans la réalisation de cet article. Principalement, le Dr Slimani Chouki qui nous a permis de réaliser cette enquête.
